# Oral Health of Australian Adults: Distribution and Time Trends of Dental Caries, Periodontal Disease and Tooth Loss

**DOI:** 10.3390/ijerph182111539

**Published:** 2021-11-02

**Authors:** Najith Amarasena, Sergio Chrisopoulos, Lisa M. Jamieson, Liana Luzzi

**Affiliations:** Australian Research Centre for Population Oral Health (ARCPOH), Adelaide Dental School, Faculty of Medical and Health Sciences, The University of Adelaide, Adelaide 5000, Australia; sergio.chrisopoulos@adelaide.edu.au (S.C.); lisa.jamieson@adelaide.edu.au (L.M.J.); liana.luzzi@adelaide.edu.au (L.L.)

**Keywords:** dental caries, oral health, periodontal disease, tooth loss

## Abstract

This study was conducted to describe the distribution and trends in dental caries, periodontal disease and tooth loss in Australian adults based on the findings of the National Study of Adult Oral Health 2017–18. A cross-sectional study of a random sample of Australians aged 15+ years was carried out, employing a three-stage stratified probability sampling design. Data were collected via online survey/telephone interviews using a questionnaire to elicit self-reported information about oral health and related characteristics. Participants were then invited to have an oral examination, conducted by calibrated dental practitioners following a standardised protocol in public dental clinics. A total of 15,731 Australians aged 15+ years were interviewed, of which 5022 dentate participants were orally examined. Results showed that nearly one third of Australian adults had at least one tooth surface with untreated dental caries and, on average, 29.7 decayed, missing or filled tooth surfaces per person. Almost 29% of adults presented with gingivitis while the overall prevalence of periodontitis was 30.1%. Overall, 4% of adults were edentulous while, on average, 4.4 teeth were lost due to pathology. Poorer oral health was evident in Australians from lower socioeconomic backgrounds, indicating socioeconomic inequalities in oral health. Time trends revealed that dental caries experience and tooth retention of Australian adults has improved over 30 years, while periodontal health has deteriorated between 2004–06 and 2017–18. These findings can be used to assist policy makers in planning and implementing future oral healthcare programs.

## 1. Introduction

Traditionally, oral health has been defined as “a state of being free from mouth and facial pain, oral diseases and disorders that limit an individual’s capacity in biting, chewing, smiling, speaking and psychosocial well-being” [1]. In view of more emphasis given to ‘absence of disease’ in this definition, a broader description for oral health has recently been suggested [2]. This broader definition advocates the definitions adopted by global and national organizations and reiterates the importance of recognising dentistry as an arena providing care and supporting oral health, rather than purely treating disease. According to the new definition, oral health is multi-faceted and includes the ability to speak, smile, smell, taste, touch, chew, swallow and convey a range of emotions through facial expressions with confidence and without pain, discomfort, and disease of the craniofacial complex, as well as being a fundamental component of general health and physical and mental wellbeing [2,3]. While proponents of this new definition aimed to reach consensus on a universal definition of oral health, this has not eventuated [4]. Global oral health aims to provide optimal oral health for all and to eradicate global health inequalities via health promotion, disease prevention and appropriate oral care strategies that incorporate common factors and resolutions, and recognise that oral health is integral to overall health [4].

Oral diseases predominantly comprise tooth decay (dental caries), gingival (periodontal) disease and oral cancers [5,6]. Dental caries, which is considered the most prevalent chronic disease worldwide [7], occur when microorganisms of dental biofilm (a sticky colourless film of bacteria build-up on the tooth surfaces) start to metabolize fermentable carbohydrates in the diet, in particular, free sugars, into acidic by-products. These acidic by-products can locally destroy (demineralise) the hard tooth structure (enamel and dentine) and initiate dental caries’ development. Frequent consumption of high free sugar and insufficient exposure to fluoride are the main contributing factors for dental caries [5,6,7]. While making enamel more resistant to acid attack, fluoride mainly acts topically to inhibit demineralisation through its presence at low concentrations in the oral fluids [5,7]. With periodontal disease, tissues that support and surround the tooth (gums, periodontal ligament and alveolar bone) are affected mainly by dental biofilm accumulated at the neck of the tooth where the tissues meet the gums (gingival margin), causing gingivitis (bleeding of the gums) [5,6]. This may lead to a more destructive form of disease, i.e., periodontitis, in susceptible individuals, particularly among those who are immunocompromised [5,6]. Poor oral hygiene, accompanied by inadequate plaque removal, is the main cause of periodontal disease, while tobacco smoking is a major risk factor associated with periodontal disease [5,6]. If left untreated, both dental caries and periodontal disease lead to tooth loss, and these two oral diseases are the major causes of tooth loss. Oral diseases affect nearly 3.5 billion people universally. Of them, approximately 2.3 billion and 530 million present with dental caries of the permanent and primary (baby) dentitions, respectively [8,9]. In 2010, severe periodontitis was ranked as the sixth-most prevalent health condition, afflicting 743 million people globally with an incidence of 701 cases per 100,000 person years [10]. Although the prevalence of severe tooth loss declined from 4.4% in 1990 to 2.4% in 2010, 158 million people worldwide were edentulous in 2010 [10].

In regard to the oral health status of Australians (based on data published prior to the National Study of Adult Oral Health 2017–18), 42% and 24% of children aged 5–10 years and 6–14 years, respectively, had experienced dental caries in their primary teeth and permanent teeth, whereas almost a quarter of Australian dentate adults aged 15 and over had untreated decay [11]. The prevalence of periodontal disease in Australian dentate adults aged 15 years and over was 22.9%, whilst 4.4% of the adult population were edentulous [11]. The present study aims at describing the distribution and time trends of dental caries, periodontal disease and tooth loss in Australian adults based on findings from the National Study of Adult Oral Health 2017–18 (NSAOH 2017–18).

## 2. Materials & Methods

Study methodology, including computation of sample size, has been described in previous studies [12,13]. Briefly, a cross-sectional study of a random sample of Australians aged 15 years and over was carried out across all Australian states and territories, employing a three-stage stratified probability sampling design. The first stage of selection included sampling of postcodes within states/territories, mainly by means of systematic sampling with probability of selection proportional to the number of households within the postcode, followed by selecting individuals aged 15 years and over within selected postcodes from the Medicare database provided by the Australian Government Department of Human Services (DHS). Accordingly, the final sample size required 15,200 interviews to be conducted, in order to complete 7200 oral examinations.

Interviews were conducted online or by telephone (CATI—computer-assisted telephone interview) by trained interviewers using a questionnaire based on previous surveys [11,14]. Self-reported information about oral health and related characteristics such as age, sex, Indigenous identity, residential location, schooling/educational qualifications, eligibility for public dental care, dental insurance and usual reason for a visit to the dentist was collected. Calibrated dental practitioners conducted oral examinations following a standardised protocol in public dental clinics run by the relevant state or territory dental health services. Inter-examiner reliability relative to a gold standard examiner was assessed by conducting replicate pairs of examination with 101 study participants. Dentate participants who consented to an examination were included for oral examinations. Although there were nine measures of oral health status, as described in detail elsewhere [15], the current analysis was confined to assessment of coronal caries (dental decay in tooth crown), gingivitis and periodontal destruction, and presence/absence of teeth (tooth loss).

### 2.1. Dental Caries (Coronal Caries)

All teeth present were subdivided into five tooth surfaces and assessed for dental caries using visual criteria without an explorer. The five tooth surfaces were mesial, buccal, distal, lingual and occlusal (for back teeth: premolars and molars)/incisal (for front teeth: incisors and canines). The mean number of decayed tooth surfaces per person denotes the severity, or burden, of untreated dental caries in people. The number of decayed, missing and filled tooth surfaces (DMFS) indicates lifetime experience of dental caries in a given person, since it has been regarded that cavities in enamel cannot heal and treatment of dental decay, either as a filling or extraction, leaves a permanent sign of disease [12].

### 2.2. Periodontal Disease

Clinical assessment for periodontal disease was conducted among those who had no medical contraindications for periodontal probing. Gingivitis and periodontitis were the two types of diseases assessed, as per the following criteria:

**Gingivitis:** Inflammation of the marginal gingival tissues around six index teeth (if present: the most anterior molar tooth in each dental quadrant + right upper central incisor + left lower central incisor) were assessed using the gingival index of Loe and Silness [16].

**Periodontitis:** The US National Health and Nutrition Examination Survey (NHANES) methods were employed to assess periodontal tissue destruction [17]. Assessments were made on three aspects (mesio-buccal, mid-buccal and disto-buccal) of all teeth present, except third molars (wisdom teeth), using a periodontal probe. To describe the prevalence of moderate and severe periodontitis, a case definition developed by the US Centers for Disease Control and Prevention (CDC) and the American Academy of Periodontology (AAP) was used [17]. Accordingly, moderate periodontitis was defined as the presence of either at least two proximal sites not on the same tooth with attachment loss of 4 mm or more, or at least two such sites that had pockets of 5 mm or more. Severe periodontitis was defined as having at least two proximal sites not on the same tooth with attachment loss of 6 mm or more, plus at least one periodontal pocket with a depth of 5 mm or more.

**Tooth loss:** Complete tooth loss (edentulism) was assessed based on the answer to the following interview question: ‘Do you have any natural teeth?’, with response categories of ‘Yes’/’No’. Existing natural teeth included crowns and caps, while dental implants were not considered natural teeth. For participants aged less than 45 years, the examiners distinguished between missing teeth that had been removed due to dental decay or periodontal disease and teeth missing due to any other reason. For participants aged 45 years or more, a removed or an absent tooth was recorded as missing.

To ensure representativeness of the target population, all data were weighted to population benchmarks [13]. Data files were managed and summary variables were computed using SAS software version 9.4 (SAS 9.4; SAS Institute Inc., Cary, NC, USA). Proportions, means and their 95% confidence were calculated where relevant.

## 3. Results

**Figure 1:**
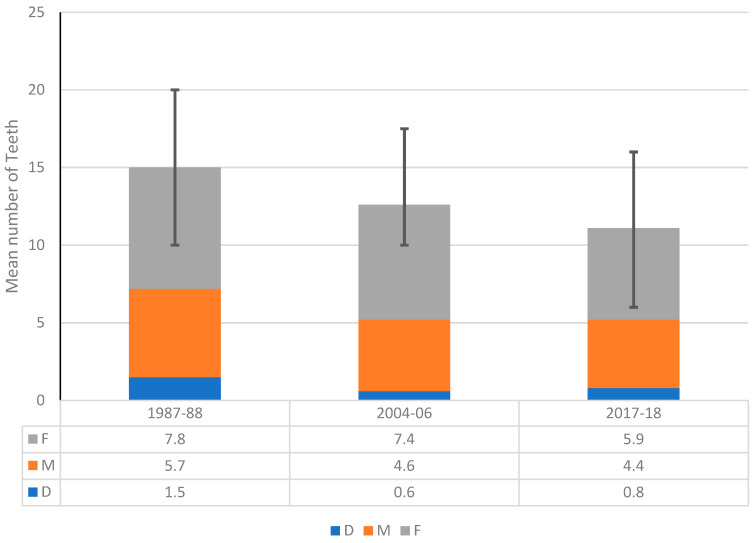
Trends in dental decay experience among dentate Australians aged 15 years and over, 1987–88, 2004–06 and 2017–18.

**Figure 2:**
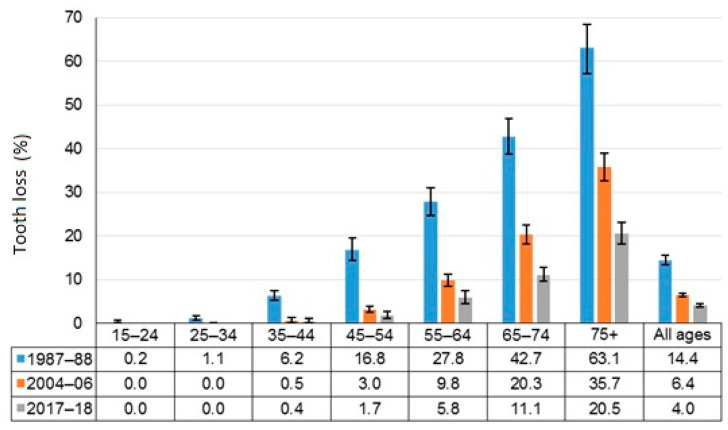
Trends in complete tooth loss among Australians aged 15 years and over, 1987–88, 2004–06 and 2017–18.

**Figure 3:**
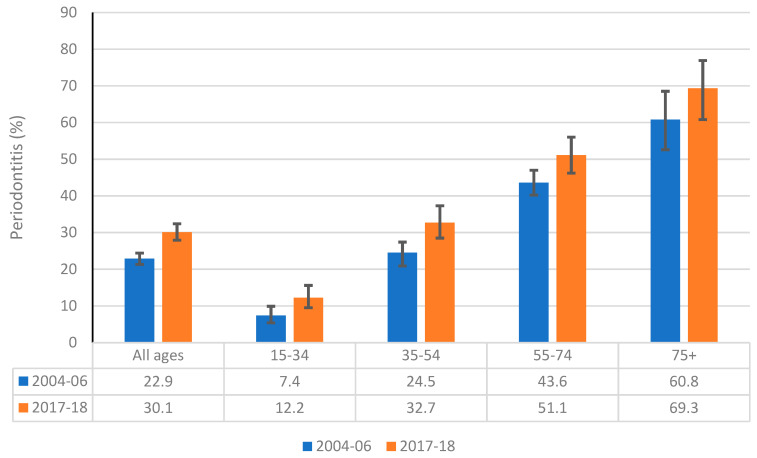
Comparison of the prevalence of moderate or severe periodontitis among dentate Australians aged 15 years and over between 2004–06 and 2017–18.

A total of 15,731 Australians aged 15 years and over completed an interview, and of them, 5022 dentate participants were orally examined. This resulted in overall participation rates of 39.7% (interview) and 33.6% (examination). Intra-class correlation coefficients (ICC) calculated to assess inter-examiner reliability were above 0.9 and 0.7 for diagnosing dental caries and periodontal disease, respectively. Weighting ensured that, approximately, an equal proportion of males (49.2%) and females (50.8) participated in the study. Given that oral health status varies considerably with age, population estimates were calculated for four age groups—15–34 years, 35–54 years, 55–74 years and ≥75 years. Table 1, Table 2, Table 3, Table 4, Table 5, Table 6 and Table 7 present the distribution of dental caries, periodontal disease and tooth loss, and Figure 1, Figure 2 and Figure 3 depict the time trends of these three conditions.

### 3.1. Dental Caries

Table 1 shows the proportion of Australian dentate adults aged 15 years and over with untreated coronal caries (one or more decayed surfaces on crowns of their teeth). Nearly one third of Australian adults (32.1%) had at least one tooth surface affected by untreated dental caries. The proportion of adults with dental caries across the four age groups varied, with the prevalence being highest in 35–54-year-olds (35.4%) and lowest among those aged 75 years and over (24.5%). The highest prevalence of untreated dental caries among participants of all ages was reported in those who visited a dentist for a dental problem (43.5%), while participants who visited the dentist for a check-up had the lowest prevalence (24.3%). Higher proportions of untreated dental caries were seen for males, those eligible for public dental care and those without dental insurance. Across age groups, higher proportions were seen for Indigenous people aged 55–74 years and those aged 35–54 years with Year 10 or less level of schooling than their counterparts.

The mean number of decayed tooth surfaces per person in Australian dentate adults aged 15 years and over is presented in Table 2. Overall, Australian dentate adults aged 15 years and over had, on average, 1.4 decayed tooth surfaces. The mean number of decayed tooth surfaces among all ages was lowest in participants who usually visited a dentist for a check-up (0.7), and usually visiting the dentist for a problem was strongly associated with higher mean number of decayed tooth surfaces across all age groups. Those who reported visiting for dental problems had, on average, 2.3 decayed surfaces. In addition, participants who had Year 11 or more schooling, a degree or higher educational qualification, those who were not eligible for public dental care and those with dental insurance had a lower mean number of decayed tooth surfaces than their counterparts.

Table 3 shows the mean number of decayed, missing or filled tooth surfaces (DMFS) per person in the Australian population. On average, Australian dentate adults aged 15 years and over had, on average, 29.7 decayed, missing or filled tooth surfaces, and it increased gradually across four age groups, with people aged ≥75 years having the highest mean DMFS (75.3). Among individuals of all ages, those who were eligible for public dental care had the highest mean DMFS (44.8), and Indigenous people had the lowest mean DMFS (18.7). Moreover, males, individuals with higher levels of schooling and degree or higher qualifications, and those who usually visited a dentist for a check-up had significantly lower mean DMFS as opposed to their counterparts.

### 3.2. Gingivitis

Table 4 shows the prevalence of gingivitis in the Australian dentate adult population. Overall, 28.8% of Australian dentate adults aged 15 years and over had gingivitis. Although the prevalence of gingivitis was decreasing with age across the four age groups, the differences were not statistically significant. Among all age groups, males had the highest prevalence of gingivitis (34.7%) and females the lowest (23.1%). In addition, those who usually visited a dentist for a dental problem (33.2%) had a greater prevalence of gingivitis than their counterparts.

### 3.3. Periodontitis

Table 5 presents the percentage of Australian dentate adults aged ≥15 years with moderate/severe periodontitis. Accordingly, the overall prevalence of moderate or severe periodontitis among the Australian dentate population was 30.1%. In contrast to gingivitis, the prevalence of moderate or severe periodontitis significantly increased with age: almost 70% of dentate adults aged ≥75 years experienced periodontitis. The prevalence of periodontitis among participants of all ages was lowest in Indigenous Australians (11.0) and highest in those participants who had Year 10 or less of schooling (45%). Males, individuals without a degree or higher qualification, those who were eligible for public dental care, those not dentally insured and those who usually visited a dentist for a dental problem experienced significantly greater periodontitis levels than their counterparts.

### 3.4. Tooth Loss

In general, 4% Australian adults aged ≥15 years had lost all their teeth (Table 6). While complete tooth loss was non-existent among the 15–34-year age group, the proportion of adults with complete tooth loss steadily increased from 1.1% among 35–54 year olds to 20.5% for those aged ≥75. Among all age groups, the dentally uninsured had the highest prevalence of complete tooth loss (10.5%), while those who with a degree or above qualification reported the lowest prevalence (0.7%). There was a subtle difference between Indigenous and non-Indigenous adults in regard to complete tooth loss, however, a significantly higher proportion of Indigenous adults aged 55–74 years reported complete tooth loss (29.3%) as opposed to their non-Indigenous equivalents (7.7%). Among all age groups, those with Year 10 or less level of schooling, those without a degree or higher qualification, people who were eligible for public dental care, the dentally uninsured and those who usually visited a dentist for a dental problem had significantly higher levels of complete tooth loss than their counterparts did.

Table 7 shows the severity of tooth loss due to pathology in Australian adults aged 15 years and over. In general, Australian adults had lost, on average, 4.4 teeth due to pathology. The mean number of teeth lost due to pathology increased consistently with age, from 0.6 at 15–34 years to 13.2 at 75 years and above. Among all age groups, the mean number of teeth lost due to pathology was lowest among those who had a degree or above qualification (2.3) and highest among those who were eligible for public dental care (7.7). In addition, people residing in rural/remote areas, those with Year 10 or less level of schooling, those dentally uninsured and those who usually visited a dentist for a dental problem had a significantly higher mean number of teeth lost due to pathology.

### 3.5. Time Trends in Oral Health

Over the past three decades, three national surveys of adult oral health have been carried out in Australia, namely, the National Oral Health Survey of Australia 1987–88 [18], the National Survey of Adult Oral Health 2004–06 [19], and the National Study of Adult Oral Health 2017–18 [12]. Accordingly, trends in oral health are sourced from these three national surveys, based on three time points. Given comparable data for periodontal disease were not available in the National Oral Health Survey of Australia 1987–88, an analysis of time trends in periodontal disease was not possible. Therefore, only a comparison of the prevalence of moderate or severe periodontitis between 2004–06 and 2017–18 surveys is presented.

Figure 1 presents the trends in the severity of dental caries experience in Australian adults aged ≥15, as denoted by mean DMFT. There has been a consistent declining trend in the mean DMFT over 30 years, from 14.9 in 1987–88 to 12.6 and 11.2 in 2004–06 and 2017–18, respectively. It was revealed that substantial reductions in all three components of the mean DMFT over 30 years have contributed to this declining trend. For example, the mean number of decayed teeth (D) and missing teeth due to pathology (M) declined from 1.5 (1987–88) to 0.8 (2017–18) and 5.7 (1987–88) to 4.4 (2017–18), respectively, whereas the average number of filled teeth (F) reduced from 7.8 in 1987–88 to 5.9 in 2017–18.

Figure 2 shows time trends in the proportion of Australian adults with complete tooth loss by age. It is apparent that there has been a steady decline in the overall proportion of Australian adults with complete tooth loss during three time points, from 14.4% in 1987–88 to 6.4% in 2004–06, and to 4% in 2017–18. This decline is reflected across all age groups, particularly among those aged 35–44 years and above, showing substantial reductions in complete tooth loss among them since 1987–88. For instance, there were only 1.7% of individuals aged 45–54 years with complete tooth loss in 2017–18, compared to 16.8% in 1987–88. The proportion of edentulous persons among 55–64-year-olds declined from 27.8% in 1987 to 5.8 in 2017–18. Likewise, nearly one in six adults aged 75+ were edentulous in 1987–88 compared to just one in three in 2017–18.

A comparison of the proportions of Australian adults with moderate or severe periodontitis by age is depicted in Figure 3. The overall prevalence of periodontal disease increased from 22.9% in 2004–06 to 30.1% in 2017–18. This was reflected in a consistent inclination of the proportion of Australian adults affected with periodontal disease across all age groups between 2004–06 and 2017–18. For example, the proportions of Australians aged 15–34 years and 75+ years with periodontitis increased from 7.4% to 12.2% and from 60.8% to 69.3, respectively, between 2004–06 and 2017–18.

## 4. Discussion

The findings of the present study indicate that overall levels of dental caries and tooth loss among Australian adults have considerably declined over the past three decades. For example, the severity of dental caries experience and complete tooth loss among Australian adults has decreased by nearly 27% and 72%, respectively, from 1987–88 to 2017–18. In general, this decline in dental caries experience has been reflected in all three components of the DMFT index, showing overall reductions of 46%, 22% and 24% in the mean number of decayed, missing and filled teeth over 30 years since 1987–88. In contrast, the periodontal status of Australian adults has substantially deteriorated between 2004–06 and 2017–18, with an overall increase in the prevalence of moderate or severe periodontitis by nearly 31%. This deterioration is evident across all age groups, in particular with the almost 65% increase in the proportion of Australian adults aged 15–34 years who have moderate or severe periodontitis.

Several factors may explain improvements in dental caries experience, as well as tooth retention, that have been observed among Australian adults over the past three decades. Nearly 90% of Australians have access to fluoridated drinking water, while almost 97% of Australian children and adults brushed their teeth daily using a fluoridated toothpaste [20]. There has been consistent evidence to suggest that community water fluoridation alongside widespread use of fluoridated toothpaste in Australia has played the most important role in preventing dental caries [21,22]. Prevention of dental caries in turn has led to increased retention of teeth, given that dental caries is regarded as the main cause of tooth loss. Furthermore, there has been a notable shift in dental treatment strategies, from high-extraction versus low-restoration to low-extraction versus high-restoration, which may have also contributed to improved tooth retention over the past three decades. These findings have consistently shown that Australian adults who usually visited only for a dental problem had higher levels of dental caries and tooth loss than those who visited for a dental check-up. For example, the severity of dental caries (as denoted by the mean DMFT) and the prevalence of complete tooth loss, respectively, were 1.31 and 6.8 times higher among Australian adults who usually visited only for a dental problem than for their counterparts who visited for a dental check-up. This finding concurs with what has been reported previously, indicating an association between improved oral health and favourable dental visiting patterns, including visiting for a dental check-up [23,24].

The NSAOH 2017–18 report has used several independent variables, such as year level of schooling, highest qualification attained, eligibility of public dental care and dental insurance, as socioeconomic indicators of the study population. Accordingly, the present findings have revealed that poor oral health has consistently been associated with lower levels of socioeconomic status. For instance, the prevalence of untreated dental decay was 1.22 times and 1.58 times higher among persons who had Year 10 or less of schooling and those who were dentally uninsured than their counterparts with Year 11 or more years of schooling and those with dental insurance. Likewise, the prevalence of complete tooth loss was 5.26 times and 3.82 times higher among individuals with Year 10 or less schooling and those who were without dental insurance, as opposed to their counterparts. These findings are consistent with those of previous studies, where more socially advantaged individuals presented with much improved oral health levels than those who were worse-off, and, consequently, supported the existence of socioeconomic inequalities in oral health [25,26].

Deterioration in periodontal health in Australian adults, which has been observed between 2004–06 and 2017–18, could be mainly ascribed to increased tooth retention. While the overall proportion of edentulous persons declined from 6.4% to 4%, the mean number of missing teeth due to pathology dropped from 4.6 to 4.4 during this period. Consequently, both the increase in the proportion of dentate adults as well as the number of retained teeth pose a greater vulnerability for periodontal disease. Our findings were consistent with those of previous studies where a strong association between age and periodontitis was observed; the older the individuals, the higher the prevalence of periodontal disease [10]. Associations between socioeconomic variables and periodontal disease, on the other hand, were similar to those seen with regard to dental caries and tooth loss. Accordingly, the prevalence of moderate or severe periodontitis was consistently higher among Australian adults in the lower socioeconomic strata. This is consistent with previous studies [10] and provides further evidence for the presence of socioeconomic disparities in oral health.

Employing a nationally representative sample of Australian adults and using a standardized examination protocol, as well as rigorous epidemiological survey methods, were some of the main strengths of the study. Other strengths included having both the interviewers and oral examiners adequately trained to ensure the quality control of the study (high intra-class correlation coefficient values were obtained indicating a high level of inter-examiner reliability and agreement), and the instruments used were based on previous studies, enabling comparisons to be made across the series of national surveys. Whilst the cross-sectional nature of the study did not warrant ascertaining cause–effect relationships, the present study could not represent Indigenous Australians in sufficient numbers. This, in turn, has resulted in creating small cell counts and relative standard errors of at least 25% in regard to Indigenous group/subgroup analyses, so interpretation of these results should be made with caution. Moreover, the use of partial recording protocols in the study could have contributed to flaws in estimating the prevalence of periodontitis. Despite such limitations, the findings showed that the overall oral health status, including the experience of dental caries, periodontal disease and tooth loss, was poorer in Indigenous Australians than in their non-Indigenous counterparts with regard to virtually all independent variables assessed. These findings are consistent with the previous studies, which were conducted among Indigenous groups in both Australia and elsewhere, indicating that Indigenous populations are among the most socioeconomically disadvantaged communities in the world [27,28,29]. It may be challenging for survey instruments and sampling methods employed in conventional population-level oral health surveys to capture the true picture of Indigenous populations and, accordingly, the need for implementing unique study methodologies for such populations has been highlighted [29].

## 5. Conclusions

The present findings suggest that the overall oral health, barring periodontal status, of Australian adults has improved over the last 30 years. Comparisons of national data between 2004–06 and 2017–18 reveal that the periodontal health of Australian adults, in general, has deteriorated during this period. The findings also indicate that individuals from lower socioeconomic backgrounds present with poorer oral health on the whole, pointing to socioeconomic inequalities in oral health. Such findings may be useful for policy makers in planning and implementing future oral healthcare programmes at a population level.

## Figures and Tables

**Table 1:** Proportion of Australian dentate adults aged 15 years and over with untreated coronal caries.

	N	% (95% CI)	Age (Years)				
			Total	15–34	35–54	55–74	≥75
			% (95% CI)				
**All people**	**5022**		**32.1** (**29.6, 34.7)**	**30.3 (25.7, 35.2)**	**35.4 (31.1, 40.0)**	**32.2 (28.2, 36.6)**	**24.5 (18.8, 31.3)**
**Sex**							
Male	2249	49.6 (46.9, 52.2)	34.7 (31.2, 38.4)	32.1 (25.1, 40.0)	37.1 (30.9, 43.7)	38.5 (33.2, 44.2)	22.3 (14.8, 32.2)
Female	2773	50.4 (47.8, 53.1)	29.5 (26.3, 32.9)	28.4 (23.2, 24.2)	33.8 (28.3, 39.8)	26.0 (20.3, 32.6)	26.2 (18.3, 36.0)
**Indigenous identity**							
Non-Indigenous	4937	98.3 (97.4, 98.9)	32.1 (29.5, 34.7)	30.6 (26.0, 35.6)	35.4 (31.1, 40.0)	31.6 (27.6, 35.9)	24.5 (18.8, 31.3)
Indigenous	84	1.7 (1.1, 2.6)	* 27.8 (15.0, 45.5)	* 17.6 (6.8, 38.3)	* 36.1(15.5, 63.4)	72.8 (45.1, 89.7)	* 22.4 (2.6, 75.9)
**Residential location**							
Major cities	2969	72.7 (69.1, 76.0)	31.8 (28.7, 35.1)	28.0 (23.4, 33.2)	35.3 (29.9, 41.2)	34.9 (29.4, 40.7)	25.7 (18.2, 34.9)
Rural/remote	2053	27.3 (24.0, 30.9)	32.6 (28.4, 37.1)	35.9 (26.0, 47.1)	35.6 (29.3, 42.6)	28.0 (22.4, 34.4)	22.5 (14.9, 32.5)
**Year level of schooling**							
Year 10 or less	1190	25.5 (23.5, 27.8)	36.9 (32.0, 42.1)	32.8 (22.4, 45.3)	51.4 (41.1, 61.6)	35.0 (28.0, 42.7)	25.3 (17.7, 34.7)
Year 11 or more	3793	74.5 (72.2, 76.5)	30.2 (27.3, 33.2)	29.6 (25.0, 34.7)	31.7 (27.1, 36.8)	29.7 (25.1, 34.9)	22.5 (14.3, 33.7)
**Highest qualification attained**							
Degree or higher	2026	29.3 (26.9, 31.8)	30.4 (26.3, 34.8)	33.1 (26.4, 40.4)	29.3 (23.6, 35.7)	27.6 (21.4, 34.7)	* 14.5 (7.7, 25.5)
Other/None	2931	70.7 (68.2, 73.1)	32.6 (29.5, 35.8)	28.6 (22.9, 35.0)	39.0 (33.2, 45.1)	32.6 (27.9, 37.6)	24.9 (18.7, 32.3)
**Eligibility for public dental care**							
Eligible	1634	30.7 (28.3, 33.1)	34.5 (30.1, 39.1)	29.3 (18.8, 42.6)	54.2 (45.5, 62.7)	32.9 (26.6, 39.8)	24.1 (18.1, 31.5)
Ineligible	3373	69.3 (66.9, 71.7)	31.1 (28.1, 34.2)	30.8 (26.0, 36.0)	31.4 (26.9, 36.3)	31.4 (26.3, 37.0)	* 26.7 (13.8, 45.4)
**Dental insurance**							
Insured	2548	45.3 (42.5, 48.1)	24.4 (21.3, 27.7)	22.3 (17.3, 28.4)	25.1 (19.8, 31.3)	25.9 (21.3, 31.1)	24.9 (17.0, 34.8)
Uninsured	2385	54.7 (51.9, 57.5)	38.6 (35.2, 42.1)	35.9 (29.8, 42.5)	45.7 (39.9, 51.6)	37.8 (31.5, 44.5)	24.3 (16.3, 34.6)
**Usually visit dentist**							
For a check-up	3135	61.5 (58.8, 64.1)	24.3 (21.4, 27.5)	24.2 (19.3, 29.9)	25.4 (20.3, 31.3)	24.4 (19.6, 30.0)	19.5 (13.1, 28.0)
For a dental problem	1796	38.5 (35.9, 41.2)	43.5 (39.3, 47.9)	43.7 (35.6, 52.2)	49.2 (42.5, 56.0)	39.4 (32.5, 46.7)	30.9 (20.1, 44.3)

* Indicates a relative standard error of at least 25%, and hence should be interpreted with caution.

**Table 2:** Mean number of decayed tooth surfaces per person in the Australian dentate adults aged 15 years and over.

	N	% (95% CI)	Age (Years)				
			Total	15–34	35–54	55–74	≥75
			Mean (95% CI)				
**All people**	**5022**		**1.4 (1.2, 1.6)**	**1.3 (0.9, 1.7)**	**1.4 (1.1, 1.7)**	**1.8 (1.3, 2.3)**	**1.1 (0.6, 1.5)**
**Sex**							
Male	2249	49.6 (46.9, 52.2)	1.7 (1.4, 2.0)	1.3 (0.8, 1.7)	1.6 (1.1, 2.1)	2.8 (1.9, 3.6)	1.1 * (0.3, 1.9)
Female	2773	50.4 (47.8, 53.1)	1.2 (0.9, 1.4)	1.4 (0.8, 1.9)	1.3 (0.9, 1.7)	0.8 (0.5, 1.0)	1.1 (0.6, 1.6)
**Indigenous identity**							
Non-Indigenous	4937	98.3 (97.4, 98.9)	1.4 (1.2, 1.6)	1.3 (0.9, 1.6)	1.4 (1.1, 1.7)	1.8 (1.3, 2.2)	1.1 (0.6, 1.6)
Indigenous	84	1.7 (1.1, 2.6)	2.7 * (0.5, 4.9)	2.5 * (0.0, 5.7)	3.2 * (0.2, 6.3)	2.8 * (0.0, 6.5)	0.9 * (0.0, 2.6)
**Residential location**							
Major cities	2969	72.7 (69.1,76.0)	1.4 (1.1,1.6)	1.3 (0.8,1.7)	1.4 (1.0,1.8)	1.6 (1.0,2.1)	1.3 (0.6,1.9)
Rural/remote	2053	27.3 (24.0,30.9)	1.6 (1.3,2.0)	1.4 (0.9,1.9)	1.5 (1.1,2.0)	2.2 (1.3,3.2)	0.7 (0.4,1.0)
**Year level of schooling**							
Year 10 or less	1190	25.5 (23.5,27.8)	2.1 (1.6,2.6)	2.6 * (1.0,4.2)	2.2 (1.5,2.9)	2.2 (1.4,3.0)	1.3 * (0.6,2.0)
Year 11 or more	3793	74.5 (72.2,76.5)	1.2 (1.0,1.4)	1.1 (0.8,1.4)	1.3 (0.9,1.6)	1.3 (0.9,1.6)	0.7 (0.4,1.0)
**Highest qualification attained**							
Degree or above	2026	29.3 (26.9,31.8)	0.9 (0.7,1.2)	0.9 (0.6,1.3)	0.9 (0.5,1.2)	1.1 (0.6,1.7)	0.9 * (0.2,1.7)
Other/None	2931	70.7 (68.2,73.1)	1.7 (1.4,1.9)	1.5 (0.9,2.1)	1.7 (1.3,2.2)	2 (1.4,2.5)	1.1 (0.5,1.6)
**Eligibility for public dental care**							
Eligible	1634	30.7 (28.3,33.1)	2.1 (1.6,2.5)	1.8 * (0.6,3.0)	2.9 (2.1,3.8)	2.3 (1.4,3.1)	1.1 (0.6,1.7)
Ineligible	3373	69.3 (66.9,71.7)	1.2 (1.0,1.4)	1.2 (0.8,1.5)	1.1 (0.8,1.4)	1.3 (0.9,1.8)	0.8 * (0.3,1.3)
**Dental insurance**							
Insured	2548	45.3 (42.5,48.1)	0.8 (0.6,1.0)	0.7 (0.4,0.9)	0.8 (0.5,1.1)	1.1 (0.7,1.6)	0.6 (0.4,0.9)
Uninsured	2385	54.7 (51.9,57.5)	1.9 (1.6,2.2)	1.6 (1.1,2.1)	2 (1.6,2.5)	2.3 (1.6,3.1)	1.4 * (0.6,2.3)
**Usually visit dentist**							
For a check-up	3135	61.5 (58.8,64.1)	0.7 (0.6,0.9)	0.7 (0.5,0.9)	0.8 (0.5,1.2)	0.6 (0.5,0.8)	0.5 (0.3,0.7)
For a dental problem	1796	38.5 (35.9,41.2)	2.3 (1.9,2.6)	2.4 (1.5,3.3)	2.1 (1.7,2.6)	2.4 (1.7,3.2)	1.8 * (0.7,2.9)

* Indicates a relative standard error of at least 25%, and hence should be interpreted with caution.

**Table 3:** Mean number of decayed, missing or filled tooth surfaces per person in Australian dentate adults aged 15 years and over.

	N	Weighted %	Age (Years)				
			Total	15–34	35–54	55–74	≥75
			Mean (95% CI)				
**All people**	**5022**		**29.7 (28.4, 31.1)**	**7.7 (6.9, 8.5)**	**24.9 (23.3, 26.5)**	**57.1 (54.8, 59.4)**	**75.3 (72.2, 78.4)**
**Sex**							
Male	2249	49.6 (46.9,52.2)	27.1 (25.2, 29.1)	7.3 (6.0, 8.5)	22.2 (19.8, 24.5)	53.5 (50.5, 56.4)	71.5 67.1, 76.0)
Female	2773	50.4 (47.8,53.1)	32.3 (30.5, 34.1)	8.1 (6.9, 9.4)	27.6 (25.6, 29.6)	60.7 (57.3, 64.2)	78.3 (74.5, 82.1)
**Indigenous identity**							
Non-Indigenous	4937	98.3 (97.4,98.9)	29.9 (28.5, 31.3)	7.7 (6.9, 8.6)	24.9 (23.3, 26.5)	57.1 (54.8, 59.5)	75.4 (72.3, 78.5)
Indigenous	84	1.7 (1.1,2.6)	18.7 (10.3, 27.1)	* 6.9 (1.1, 12.7)	27.5 (22.7, 32.3)	63.9 (54.9, 72.8)	NP
**Residential location**							
Major cities	2969	72.7 (69.1,76.0)	28.5 (26.9, 30.1)	7.8 (6.8, 8.9)	24.4 (22.5, 26.3)	57 (53.6, 60.4)	77.8 (73.8, 81.7)
Rural/remote	2053	27.3 (24.0,30.9)	32.3 (29.8, 34.8)	7.3 (5.9, 8.8)	26 (23.1, 28.9)	57.2 (54.7, 59.7)	71.1 (66.9, 75.4)
**Year level of schooling**							
Year 10 or less	1190	25.5 (23.5,27.8)	43.9 (41.1–46.8)	7.6 (4.6, 10.6)	29.9 (26.1, 33.8)	57 (53.4, 60.7)	75.6 (71.2, 80.0)
Year 11 or more	3793	74.5 (72.2,76.5)	24.8 (23.4, 26.2)	7.6 (6.8, 8.5)	23.9 (22.1, 25.6)	57.3 (54.3, 60.3)	74.8 (71.2, 78.4)
**Highest qualification attained**							
Degree or higher	2026	29.3 (26.9,31.8)	20.9 (19.2, 22.5)	8.4 (7.2, 9.6)	19 (17.1, 20.8)	55.8 (52.9, 58.7)	76.3 (72.0, 80.6)
Other/None	2931	70.7 (68.2,73.1)	33.4 (31.8, 35.1)	7.3 (6.2, 8.4)	28.3 (26.1, 30.5)	58 (55.4, 60.5)	75.7 (72.1, 79.2)
**Eligibility for public dental care**							
Eligible	1634	30.7 (28.3,33.1)	44.8 (42.0, 47.6)	8.8 (6.8, 10.7)	32.5 (28.6, 36.4)	58.9 (55.2, 62.5)	75.5 (72.0–79.0)
Ineligible	3373	69.3 (66.9,71.7)	23.2 (21.8, 24.5)	7.5 (6.6, 8.4)	23.3 (21.6, 25.0)	55.4 (52.5, 58.3)	74.1 (69.2, 79.0)
**Dental insurance**							
Insured	2548	45.3 (42.5,48.1)	30.6 (28.8, 32.4)	7.5 (6.2, 8.7)	23.2 (21.1, 25.4)	59.4 (57.0, 61.8)	76.4 (72.8, 79.9)
Uninsured	2385	54.7 (51.9,57.5)	29.9 (27.9, 31.8)	7.9 (6.8, 9.1)	27.1 (24.6, 29.5)	55.3 (51.6, 59.0)	74.6 (69.8, 79.3)
**Usually visit dentist**							
For a check-up	3135	61.5 (58.8,64.1)	26.6 (25.0, 28.1)	6.5 (5.6, 7.3)	21.9 (19.8, 23.9)	56.7 (54.3, 59.2)	75.5 (71.1, 79.8)
For a dental problem	1796	38.5 (35.9,41.2)	35.7 (33.4, 37.9)	10.8 (8.9, 12.7)	29.8 (27.2, 32.4)	57.7 (53.5, 61.9)	75.3 (71.1, 79.4)

* Indicates a relative standard error of at least 25%, and hence should be interpreted with caution. NP: Not publishable due to small cell counts.

**Table 4:** Percentage of people with gingival inflammation in the Australian dentate population.

	N	% (95% CI)	Age (Years)				
			Total	15–34	35–54	55–74	≥75
			% (95% CI)				
**All people**	**4401**		**28.8 (26.1, 31.6)**	**31.3 (27.1, 35.8)**	**29.5 (25.2, 34.2)**	**24.4 (20.7, 28.6)**	**20.9 (15.0, 28.2)**
**Sex**							
Male	1906	48.9 (46.0,51.8)	34.7 (30.7,39.0)	34.9 (28.5,41.8)	35.6 (29.3,42.4)	34.1 (28.0,40.8)	27.4 (17.0,41.1)
Female	2496	51.1 (48.2,54.0)	23.1 (20.3,26.1)	27.6 (22.7,33.0)	23.7 (18.8,29.3)	15.7 (12.1,20.3)	16.7 (10.3,26.0)
**Indigenous identity**							
Non-Indigenous	4330	98.4 (97.4,99.0)	28.7 (26.0,31.5)	31.3 (27.0,35.9)	29.1 (24.8,33.8)	24.6 (20.8,28.9)	20.9 (15.1,28.2)
Indigenous	71	1.6 (1.0,2.6)	38.6 (19.9,61.4)	30.5 * (11.1,60.7)	63.3 (36.2,84.0)	9.9 * (1.2,49.2)	NP
**Residential location**							
Major cities	2607	73.8 (70.3,77.0)	30.1 (26.8,33.5)	31.5 (26.8,36.6)	31.6 (26.2,37.5)	26.0 (21.0,31.7)	21.9 (14.9,30.8)
Rural/remote	1795	26.2 (23.0,29.7)	25.2 (20.6,30.4)	30.4 (21.8,40.6)	24.2 (18.1,31.4)	21.1 (16.3,26.9)	18.2 * (9.2,32.9)
**Year level of schooling**							
Year 10 or less	943	23.2 (21.2,25.4)	28.6 (24.0,33.8)	40.2 (27.5,54.3)	30.4 (22.1,40.1)	23.3 (17.5,30.2)	18.6 (11.4,28.8)
Year 11 or more	3427	76.8 (74.6,78.8)	28.9 (25.9,32.1)	29.9 (25.5,34.7)	29.3 (24.6,34.6)	25.6 (21.0,30.9)	24.5 (15.7,36.0)
**Highest qualification attained**							
Degree or above	1865	30.6 (28.1,33.3)	24.0 (20.5,28.0)	21.3 v	27.3 (21.2,34.3)	24.2 (17.5,32.5)	22.2 * (11.3,39.1)
Other/None	2477	69.4 (66.7,71.9)	31.2 (27.9,34.6)	36.7 (31.1,42.7)	31.0 (25.6,37.0)	25.2 (20.9,30.0)	20.7 (14.1,29.3)
**Eligibility for public dental care**							
Eligible	1264	27.3 (24.9,29.9)	30.4 (26.0,35.3)	31.9 (22.8,42.5)	38.1 (29.1,48.0)	28.4 (22.2,35.5)	19.6 (13.5,27.6)
Ineligible	3123	72.7 (70.1,75.1)	28.3 (25.3,31.5)	31.3 (26.7,36.4)	27.8 (23.3,32.8)	21.4 (17.0,26.4)	29.6 * (14.1,51.8)
**Dental insurance**							
Insured	2261	46.1 (43.0,49.2)	25.2 (22.0,28.8)	29.9 (24.0,36.5)	25.0 (19.8,31.1)	20.4 (16.2,25.3)	14.9 (9.1,23.6)
Uninsured	2058	53.9 (50.8,57.0)	31.1 (27.5,34.9)	30.1 (24.6,36.1)	34.8 (28.6,41.6)	28.4 (22.5,35.1)	25.8 (16.9,37.3)
**Usually visit dentist**							
For a check-up	2775	62.3 (59.5,65.1)	25.2 (22.0,28.7)	27.5 (22.5,33.1)	25.2 (20.2,31.1)	20.7 (16.7,25.3)	20.5 (13.6,29.9)
For a dental problem	1548	37.7 (34.9,40.5)	33.2 (29.3,37.4)	35.4 (27.9,43.6)	35.7 (29.3,42.8)	28.7 (22.6,35.8)	21.4 * (12.2,34.9)

* Indicates a relative standard error of at least 25%, and hence should be interpreted with caution. NP: not publishable due to small cell counts.

**Table 5:** Proportion of people with moderate or severe periodontitis in the Australian dentate population.

	N	% (95% CI)	Age (Years)				
			Total	15–34	35–54	55–74	≥75
			% (95% CI)				
**All people**	**4402**		**30.1 (27.9, 32.4)**	**12.2 (9.5, 15.6)**	**32.7 (28.5, 37.3)**	**51.1 (46.2, 56.0)**	**69.3 (60.5, 76.9)**
**Sex**							
Male	1906	48.9 (46.0,51.8)	34.9 (31.2,38.8)	16.6 (11.8,22.8)	38.9 (32.1,46.3)	59.5 (53.3,65.4)	63.1 (48.1,75.9)
Female	2496	51.1 (48.2,54.0)	25.5 (22.7,28.5)	7.8 (5.3,11.3)	26.6 (21.7,32.2)	43.5 (37.1,50.2)	73.0 (62.3,81.6)
**Indigenous identity**							
Non-Indigenous	4330	98.4 (97.4,99.0)	30.3 (28.1,32.7)	12.5 (9.7,15.9)	32.9 (28.6,37.5)	50.8 (46.0,55.6)	69.2 (60.4,76.8)
Indigenous	71	1.6 (1.0,2.6)	11.0 * (5.3,21.3)	3.9 * (0.8,17.2)	21.0 * (8.2,44.1)	49.7 * (15.4,84.3)	NP
**Residential location**							
Major cities	2607	73.8(70.3,77.0)	29.4 (26.7,32.2)	12.2 (9.0,16.4)	31.6 (26.4,37.2)	52.9 (46.7,59.1)	71.1 (60.1,80.0)
Rural/remote	1795	26.2 (23.0,29.7)	32.1 (28.1,36.5)	12.3 (8.2,18.2)	35.8 (28.8,43.4)	47.1 (39.6,54.8)	64.4 (49.7,76.9)
**Year level of schooling**							
Year 10 or less	943	23.2 (21.2,25.4)	45.0 (39.6,50.5)	7.7 * (3.3,16.7)	50.0 (39.8,60.3)	55.9 (47.8,63.7)	72.2 (61.0,81.1)
Year 11 or more	3427	76.8 (74.6,78.8)	25.6 (23.2,28.2)	12.9 (9.8,16.8)	29.2 (24.8,34.1)	47.8 (42.3,53.3)	64.7 (49.2,77.7)
**Highest qualification attained**							
Degree or above	1865	23.2 (21.2,25.4)	21.7 (18.2,25.6)	11.6 (6.7,19.1)	22.7 (18.1,28.1)	49.7 (42.6,56.7)	59.6 (35.9,79.6)
Other/None	2477	69.4 (66.7,71.9)	33.6 (30.6,36.6)	12.6 (9.5,16.5)	38.4 (32.5,44.6)	50.9 (45.3,56.5)	69.9 (60.6,77.8)
**Eligibility for public dental care**							
Eligible	1264	27.3 (24.9,29.9)	42.5 (37.9,47.2)	15.7 (9.0,25.9)	41.3 (32.1,51.2)	54.8 (47.5,61.9)	70.6 (61.5,78.3)
Ineligible	3123	72.7 (70.1,75.1)	25.5 (22.9,28.2)	11.5 (8.7,14.9)	30.9 (26.2,36.0)	47.7 (41.8,53.7)	59.3 (33.3,80.9)
**Dental insurance**							
Insured	2261	46.1 (43.0,49.2)	25.4 (22.7,28.3)	8.4 (5.1,13.4)	24.5 (19.8,30.0)	45.2 (39.0,51.6)	67.4 (53.1,79.1)
Uninsured	2058	53.9 (50.8,57.0)	35.0 (31.8,38.4)	15.7 (11.5,20.9)	41.1 (34.8,47.7)	56.9 (49.7,63.8)	70.7 (59.7,79.8)
**Usually visit dentist**							
For a check-up	2775	62.3 (59.5,65.1)	26.1 (23.4,29.0)	8.8 (6.0,12.9)	29.5 (23.9,35.8)	49.0 (43.0,55.0)	72.5 (60.4,81.9)
For a dental problem	1548	37.7 (34.9,40.5)	36.8 (32.6,41.3)	18.8 (13.4,25.8)	37.2 (30.4,44.5)	53.0 (45.6,60.2)	64.3 (50.3,76.2)

* Indicates a relative standard error of at least 25%, and hence should be interpreted with caution. NP: not publishable due to small cell counts.

**Table 6:** Proportion of adults with complete tooth loss in the Australian population.

	N	% (95% CI)	Age (Years)				
			Total	15–34	35–54	55–74	≥75
			% (95% CI)				
**All people**	**15,731**		**4.0 (3.6, 4.4)**	**—**	**1.1 (0.7, 1.6)**	**8.1 (7.0, 9.3)**	**20.5 (18.1, 23.1)**
**Sex**							
Male	6781	49.2 (48.1,50.4)	3.4 (2.9,3.9)	—	1.1 * (0.6,2.0)	6.5 (5.2,8.1)	19.1 (15.6,23.2)
Female	8950	50.8 (49.6,51.9)	4.7 (4.1,5.3)	—	1.0 * (0.6,1.8)	9.6 (8.0,11.5)	21.5 (18.4,25.0)
**Indigenous identity**							
Non-Indigenous	15,392	97.7 (97.3,98.1)	4.0 (3.6,4.4)	—	1.1 (0.7,1.6)	7.7 (6.7,8.9)	20.5 (18.1,23.1)
Indigenous	334	2.3 (1.9,2.7)	7.1 (4.3,11.4)	—	0.8 * (0.2,2.5)	29.3 (17.8,44.1)	19.5 * (6.5,45.9)
**Residential location**							
Major cities	9372	71.8 (68.6,74.9)	3.5 (3.0,4.0)	—	1.0 * (0.6,1.7)	7.4 (6.0,9.0)	18.8 (15.9,22.0)
Rural/remote	6359	28.2 (25.1,31.4)	5.4 (4.7,6.2)	—	1.2 * (0.7,2.0)	9.5 (8.1,11.2)	24.2 (20.1,28.7)
**Year level of schooling**							
Year 10 or less	4198	28.9 (27.8,30.1)	9.4 (8.5,10.5)	—	3.1 * (1.8,5.2)	11.7 (9.9,13.8)	24.9 (21.6,28.5)
Year 11 or more	11,355	71.1 (69.9,72.2)	1.8 (1.5,2.1)	—	0.6 * (0.3,1.1)	5.3 (4.2,6.7)	13.1 (10.2,16.6)
**Highest qualification attained**							
Degree or higher	5836	26.8 (25.4,28.2)	0.7 (0.5,1.1)	—	0.5 * (0.1,1.6)	2.0 (1.3,3.1)	5.3 * (3.0,9.0)
Other/None	9584	73.2 (71.8,74.6)	5.1 (4.6,5.7)	—	1.3 (0.8,2.0)	9.4 (8.1,10.8)	22.0 (19.4,24.9)
**Eligibility for public dental care**							
Eligible	4976	30.2 (29.0,31.4)	10.5 (9.5,11.7)	—	3.1 * (1.7,5.3)	13.4 (11.5,15.6)	22.3 (19.6,25.2)
Ineligible	10,686	69.8 (68.6,71.0)	1.2 (1.0,1.5)	—	0.7 * (0.4,1.2)	3.7 (2.9,4.9)	11.3 (7.6,16.5)
**Dental insurance**							
Insured	8238	51.1 (49.5,52.8)	1.7 (1.4,2.0)	—	0.5 * (0.3,1.1)	3.6 (2.8,4.5)	9.2 (7.0,11.9)
Uninsured	7206	48.9 (47.2,50.5)	6.5 (5.8,7.2)	—	1.8 (1.1,2.8)	12.7 (10.9,14.8)	28.3 (24.7,32.3)
**Usually visit dentist**							
For a check-up	9790	63.3 (61.9,64.6)	1.2 (0.9,1.5)	—	0.3 * (0.2,0.6)	3.0 (2.1,4.2)	6.1 (4.4,8.4)
For a dental problem	5620	36.7 (35.4,38.1)	7.9 (7.1,8.8)	—	2.2 (1.3,3.5)	13.0 (11.2,15.0)	32.5 (28.5,36.7)

* Indicates a relative standard error of at least 25%, and hence should be interpreted with caution.

**Table 7:** Mean number of missing teeth for pathology per person in the Australian dentate population.

	N	% (95% CI)	Age (Years)				
			Total	15–34	35–54	55–74	≥75
			Mean (95% CI)				
**All people**	**5022**		**4.4 (4.1, 4.7)**	**0.6 (0.4, 0.7)**	**3.6 (3.3, 3.9)**	**8.8 (8.2, 9.4)**	**13.2 (12.2, 14.2)**
**Sex**							
Male	2249	49.6 (46.9,52.2)	4.2 (3.8,4.6)	0.5 (0.3,0.8)	3.4 (3.0,3.9)	8.6 (8.0,9.3)	13.6 (12.5,14.6)
Female	2773	50.4 (47.8,53.1)	4.6 (4.2,5.0)	0.7 (0.4,0.9)	3.8 (3.4,4.2)	9 (8.0,10.0)	12.9 (11.3,14.6)
**Indigenous identity**							
Non-Indigenous	4937	98.3 (97.4,98.9)	4.4 (4.1,4.7)	0.6 (0.4,0.7)	3.6 (3.3,3.9)	8.8 (8.2,9.4)	13.2 (12.2,14.2)
Indigenous	84	1.7 (1.1,2.6)	3.2 (1.6,4.7)	0.9 * (0.0,1.7)	4.9 (3.2,6.7)	11.5 (7.0,16.0)	14 * (1.9,26.0)
**Residential location**							
Major cities	2969	72.7 (69.1,76.0)	4 (3.7,4.4)	0.6 (0.4,0.8)	3.4 (3.0,3.8)	8.4 (7.6,9.2)	13.3 (12.0,14.6)
Rural/remote	2053	27.3 (24.0,30.9)	5.4 (4.9,5.9)	0.7 (0.4,1.0)	4.3 (3.7,4.8)	9.6 (8.8,10.3)	13 (11.6,14.4)
**Year level of schooling**							
Year 10 or less Year 11 or more	1190 3793	25.5 (23.5,27.8) 74.5 (72.2, 76.5)	7.7 (7.1,8.2) 3.3 (3.0, 3.5)	0.6 * (0.2,1.0) 0.6 (0.4, 0.7)	4.7 (4.0,5.4) 3.4 (3.0, 3.8)	10.2 (9.4,11.1) 7.7 (6.8, 8.6)	14 (12.5,15.6) 11.8 (10.7, 12.9)
**Highest qualification attained**							
Degree or above	2026	29.3 (26.9,31.8)	2.3 (2.0,2.5)	0.6 (0.3,0.8)	2.4 (2.0,2.7)	6 (5.4,6.6)	11 (9.1,13.0)
Other/None	2931	70.7 (68.2,73.1)	5.3 (4.9,5.6)	0.6 (0.4,0.8)	4.3 (3.9,4.8)	9.4 (8.7,10.1)	13.4 (12.2,14.5)
**Eligibility for public dental care**							
Eligible	1634	30.7 (28.3,33.1)	7.6 (7.0,8.2)	1 (0.5,1.4)	5.2 (4.5,6.0)	10.1 (9.3,11.0)	13.6 (12.5,14.7)
Ineligible	3373	69.3 (66.9,71.7)	3 (2.7,3.3)	0.5 (0.4,0.6)	3.3 (2.9,3.6)	7.6 (6.7,8.4)	10.8 (9.1,12.5)
**Dental insurance**							
Insured	2548	45.3 (42.5,48.1)	3.9 (3.5,4.2)	0.4 (0.3,0.6)	3.0 (2.6,3.4)	7.6 (7.0,8.3)	10.8 (9.8,11.8)
Uninsured	2385	54.7 (51.9,57.5)	5 (4.6,5.4)	0.7 (0.5,1.0)	4.3 (3.8,4.8)	9.8 (9.0,10.7)	15 (13.4,16.5)
**Usually visit dentist**							
For a check-up	3135	61.5 (58.8,64.1)	3.5 (3.2,3.8)	0.5 (0.3,0.7)	3.1 (2.7,3.5)	7.3 (6.8,7.9)	11.3 (10.3,12.3)
For a dental problem	1796	38.5 (35.9,41.2)	6 (5.5,6.5)	0.8 (0.6,1.1)	4.5 (4.0,5.0)	10.6 (9.5,11.7)	16 (14.2,17.8)

* Indicates a relative standard error of at least 25%, and hence should be interpreted with caution.

## Data Availability

The datasets used during the current study are available from the corresponding author via completion of a data request.
